# 

**Published:** 1889-02-02

**Authors:** 


**Notice to Advertisers and Subscribers.**

All Advertisements, Orders for Copies of The Hospital, and Business Communications should be addressed to The Publisher, *not to the Editor*, The Hospital, 38, Old Jewry, E.C.

A Scale of Charges will be found on the first page of the Nursing Supplement.

**The Hospital Annual!** Great difficulty has been experienced in securing the return of the schedule of particulars from certain institutions, despite three applications to the authorities. This has caused an unexpected delay in the issue of the Annual. It has therefore been determined to go to press next week, and to insert a notification to that effect when the schedules have not been returned. Everyone will thus be left to draw their own conclusions, and those who suffer from their own neglect will have no reasonable ground of complaint.

### Scraps and Gleanings.

Canon Larkin is now performing faith cures at Glasgow.

Dr. Magner has been elected physician to Cork North Infirmary.

There were 5,000 cases of infectious diseases in Edinburgh last year.

Nearly 700 patients were treated last year by the Hastings Free Dispensary.

At Pendlebury Children's Hospital only 114 out of the 140 beds are now occupied.

Colonel North has offered £500 towards a cottage hospital for Glamorganshire.

The Executive Committee of the General Medical Council will meet on February 25th.

Barrington's Hospital, Limerick, has entered upon the fifty-eighth year of its existence.

Count Tolsti, the Russian novelist, is now heading a temperance movement in Moscow.

The Gorleston Cottage Hospital requires £600, of which £350 has been already subscribed.

The M'Lean Hospital, Dunfermline, is to be reconstructed for infectious cases, at a cost of £1,128.

The last meeting of the old Cottage Hospital Committee at Bournemouth was held on January 18th.

Mr. Ernest Hart is writing a series of interesting letters on "Medical Paris" to the *British Medical Journal.*

The Sunderland Hospital Committee have visited Walker to inspect the furnishing and fittings of that hospital.

A ball was given last week at Brighton in aid of that excellent institution, St. John's Convalescent Home for Children.

The Medical Society when at Leeds this year is to be invited to inspect the new Bath Hospital and Convalescent Home at Harrogate.

The Brighton, Hove, and Preston Dispensary issues a satisfactory report, thanks to the noble munificence of Mrs. Carr-Burton.

The first insane asylum in Greece was recently established in Attica. Hitherto the insane in that country have been cared for in convents.

The Urban Sanitary Authority have protested against the closing of Londonderry Fever Hospital, which has been in existence thirty-six years.

Mr. Donald MacLeod, chief attendant at Glasgow District Asylum, has been awarded the Morison prize for meritorious attendance on the insane.

You can be cremated in Philadelphia for £5 5s., but the inhabitants don't appreciate the privilege, and the crematory is likely to prove a failure.

Forres Cottage Hospital has had a further munificent donation of £3,000 from the anonymous friend who formerly subscribed so handsomely.

The Misses Wynne, of Hazlewood, have fitted up a ward in the County Sligo Infirmary as a memorial of their late father, the Right Hon. John Wynne.

Mrs. Drew and Miss Wall (matron) organised a splendid concert on January 19th for the inmates of St. Martin's Ophthalmic Hospital, Dublin.

At the late meeting of the Nottingham Hospital Saturday Committee, the Rev. H. Seymour favoured the audience with a number of medical anecdotes.

The Christmas entertainment at the Manchester Clinical Hospital was a very pleasant one, thanks to Miss Tylor, the matron, and Mr. Walsh, house surgeon.

The Glasgow Convalescent Home, at Lenzie, continues to do good work. 1,498 patients were treated last year, at the cost of 1s. 4d. per patient per day.

The house-surgeon of Bridgenorth and South Shropshire Infirmary reports that last quarter there was a diminution of illness in the town, and no epidemic.

A correspondent writes to the *Liverpool Mercury* to point out that the Welsh places of worship are behindhand in subscribing to the Hospital Sunday Fund.

The proposal to abolish Derry Fever Hospital needs further consideration; respectable people would not go to the workhouse infirmary if there were an epidemic.

The Queen has sent her "Leaves From Our Journal in the Highlands," and "More Leaves," each with autograph inscription, to the new Birmingham Infirmary.

A correspondent of the *Essex Telegraph* comments on the excellent management of the Eastern Counties Asylum, where constant entertainments are provided for the inmates.

Hospital Sunday at Liverpool apparently did well, but the result will not be known for a short time. The collection at Sefton Park Presbyterian Church reached £581, and at Renshaw Street (Unitarian) £533.

A fearful epidemic of diphtheria is reported from the town of Nago, in Hungary. Children have been dying during the last week at the rate of twenty a day. All the schools have been closed, and there is a panic among the inhabitants.

Mr. W. G. Bagnall, at the recent general meeting of the Governors of the Staffordshire General Infirmary, advised that patients should be discharged at the discretion of the medical staff, instead of waiting for a meeting of the hospital committee.

Earl Stanhope presided at the annual meeting of the Holmesdale Cottage Hospital at Sevenoaks on the 29th ult., and spoke of the important advantage which the institution, which was founded on the provident system, offered to the members of the industrial classes.

With the assistance of the Earl of Radnor the authorities at Folkestone have decided upon a site for a new hospital near Radnor Park. The hospital, which is to be a memorial of the Queen's Jubilee, will be a very handsome building, and nearly £14,000 has been subscribed towards the cost.

### Amusements and Relaxation.

### NEW PUZZLE PRIZE SCHEME.

SECOND QUARTERLY COMPETITION COMMENCED JANUARY 5th, ENDS MARCH 30th, 1889.

1. Prizes will be awarded for the best Original Puzzles in Prose or Verse relating to any of the Social, Political, or Philanthropical topics of the day. Competitors are requested to *strictly* observe this rule, or their contributions will be discredited.
2. Puzzles may take any form which the ingenuity of the competitors suggest. Answers must accompany contributions, and the source of obscure or ambiguous terms be given.
3. No competitor will be allowed to take more than one prize during the Quarter.
4. Eight Prizes of Standard Books will be given, the choice of the book to be left to the winner. First, Second, Third, Fourth, and Fifth Prizes for best Original Puzzles, £1 1s., 15s., 10s. 6d., 7s. 6d., and 5s. each.
5. First, Second, and Third Prize for Solutions of 15s., 10s. 6d., and 5s. each.

N.B.—All contributions must reach the office, 38, Old Jewry, E.C., not later than the *First Post* on *Thursday* in each week.

### SPECIAL NOTICE.

N.B.—The Prize Editor regrets that more competitors have not entered for the present quarter. Doubtless the holidays may have something to do with this, and he trusts that all intending competitors will forward their contributions without further delay; and that they, with those who have already entered the lists, will call the attention of their puzzle-making friends to this column of the paper, which, from its retired position, is unknown to many of the numerous readers of The Hospital.

### PUZZLES FOR SOLUTION.

**Fifth Week.**

No. 10. Arithmorem.

1. O B. 1000 (and) Orange—a wooden missile.
2. A. 501 (and) Ago—a slow movement in music.
3. G T. 52 (and) Yarns—an inflammation.
4. R. 100 (and) Fan—a coin.
5. O. 1105 (and) Heart—too powerful.
6. E. 150 (and) Un—a near relation.
7. R. 7 (and) Nose—a correction.

Initials and finals downwards name two well-known statesmen.

Morico.

No. 11. Original Anagram.

Deliver! Help! for oft our city innocents cry to thee!

Cotonopolis.

**Marks for Original Puzzles.**

| Names. | No. of Puzzles sent in Jan. 24. | No. of Marks Jan. 24. | Totals. | Names. | No. of Puzzles sent in Jan. 24. | No. of Marks Jan. 24. | Totals. |
|---|---|---|---|---|---|---|---|
| Cotonopolis... | 3 | 4 | 16 | Padre ......... | — | — | 3 |
| Tinie............ | 3 | 5 | 16 | Prior............ | 3 | 3 | 7 |
| Jenny Wren . | — | — | 4 | Lightowlers . | 1 | 2 | 4 |
| Patience ...... | 3 | 4 | 15 | Twentieth ... | — | — | 3 |

**Answers.**

No. 6. Double Acrostic.

| | | |
|---|---|---|
| A | lexande | R |
| M | nemosyn | E |
| U | ra | L |
| S | taffa | A |
| E | sse | X |
| M | aria Theres | A |
| E | llio | T |
| N | icolin | I |
| T | ass | O |
| S | olution | N |

No. 7. Wheel Puzzle.

| | | |
|---|---|---|
| 1. S | Salop | P |
| 2. U | Ulema | A |
| 3. A | Aspen | N |
| 4. K | Khama | A |
| 5. I | Idiom | M |
| 6. M | Medea | A |

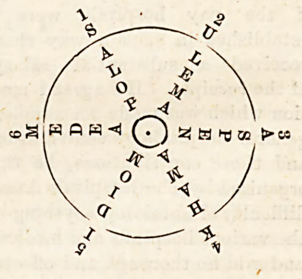

**Marks for Solution of Puzzles.**

Total Marks.—Flora (1), 2; Mater, 0; Scrooge (1), 2; Patience 0; Lady Clara (1), 3; Ruffles (1), 2; Lauriston, 0; Reldas, 0; Jenny Wren, (1) 2.

[*The Figures in parentheses are Marks for the week ending Jan. 24th.*]

**Conditions.**

I. Every paper sent in must be clearly written, and one side only must be used. All competitors must mark at the head of their papers the number of their competition.

II. Each paper must be signed by the author with his or her real name and address. A *nom de plume* may be added if the writer does not desire to be referred to by us by his real name. In the case of all prizewinners, however, the real name and address will be published.

III. All communications relating to prize competitions should be addressed to "The Prize Editor, The Hospital, 38, Old Jewry, London, E.C." Competitors are requested not to include in letters, etc., to the Prize Editor, communications on matters of other business. All letters must be fully prepaid.

IV. The Prize Editor of The Hospital is the sole judge of the competitions, and his decision is in all cases final and without appeal.

V. The Prize Editor reserves the right to publish any paper sent in, whether it receives a prize or not. He does not hold himself responsible for any MSS. sent, nor can he undertake to return rejected competitions.

